# "Peter Pan" in a Private Mental Hospital

**Published:** 1914-01-03

**Authors:** 


### "Peter Pan" in a Private Mental Hospital.

For some weeks past there has been an undercurrent of quiet preparation. Although the decorations throughout the house appeared to have sprung into existence in a single night—up to Christmas Eve they apparently were not—yet many hours of work have been necessary for their production. The nurses have been employed in the fabrication of these decorations, not only at intervals during the day but also, it is evident, they have given up much of their off-duty time in order that their ward should not be lacking in adornment; and in this work they have been loyally seconded by the patients, into whom also the spirit of laudable emulation has been infused. The hyper-artistic being recoils from the vision of what he considers an anathema by his meticulous canons of criticism—the substitution of plaster, for instance, in place of bronze or marble in a statuette, or of artificial flower and leaf for the reality. Yet even his censorious mind might have experienced some feeling of æsthetic gratification had he observed the results of these labours. From sundry rolls of coloured paper had been fabricated by deft fingers really admirable counterfeits of roses, of clematis, of almond blossom, and of other flowers. In one ward singularly realistic sprays of broom were to be seen, the flowers attached to pieces of shrubs obtained for the purpose from the gardens. All of these tended to give a cheerful brightness which holly and evergreens cannot impart, although these were discreetly mingled with the other decorations.

On Christmas Day we assembled in the morning to sing carols at the Morning Service and to listen to the words of wisdom and of safe counsel which our chaplain addressed to us. Then came the characteristic function by which all English people are known to celebrate their festivals—a feast! Turkey and plum-pudding—without which so many would reckon Christmas inefficiently observed. "Trimmins," of course, were not wanting, but they naturally fill but a subsidiary position—literally and metaphorically. After a due interval for contemplation—and more or less efficient digestion—we all met in the large recreation room for our Christmas-tree. It was not exactly a tree. There were a number of trees, and embowered amongst them was an extremely large doll's house, representing that well-known scene in Barrie's delightful play "Peter Pan," the "House in the Tree Tops." The house was placed upon a wooden platform some six feet high, and the woodwork was almost entirely obscured by a number of small Christmas-trees and shrubs, the whole giving quite a realistic effect. And the trees were ornamented as Christmas-trees are wont to be ornamented, with glittering things and tinsel and little lanterns. In the house were numerous presents which were handed down from the tree-tops by Peter in a real Peter's costume—the medical superintendent's little daughter, who played her part delightfully. Then there was tea and talk; and as the patients who were present were in almost equal numbers of either sex, there was a good deal of talk. (Even the most militant male or female will find it difficult to criticise that ambiguous statement!) And after tea back to the wards again for the little extra bits of amusement which had been provided to round off the day. In the infirmary, for instance, wherefrom the patients could not go to the general entertainment, the nurses had provided a "bran-pie," into which you had to make a "dip" in order to extract one of the little packages. There were packets of sweets and little black, weird, and woolly dogs, and all sorts of odd things. And on top of the pie before it was opened was the little presiding fairy, a golden-haired doll bespangled and glittering. The staff were tired with the extra labours which the festivities have entailed, but pleased that they have achieved some success in driving dull care away from our "Asylum Christmas."

